# Image-based modeling of gas adsorption and deformation in porous media

**DOI:** 10.1038/s41598-018-26197-8

**Published:** 2018-05-29

**Authors:** Sahar Bakhshian, Zhuofan Shi, Muhammad Sahimi, Theodore T. Tsotsis, Kristian Jessen

**Affiliations:** Mork Family Department of Chemical Engineering and Materials Science, University of Southern California, Los Angeles, California 90089-1211 USA

## Abstract

Understanding adsorption of CO_2_ in porous formations is crucial to its sequestration in geological formations. We describe a model for adsorption of CO_2_ and the deformation that it induces in a sandstone formation over wide ranges of temperature and pressure. The model couples the thermodynamics of sorption with elastic deformation of the solid. Finite-element computations are then used in order to compute CO_2_ adsorption isotherms along with the induced strain in the formation. We also compute the Darcy permeability of the porous medium using the lattice-Boltzmann method. All the computations are carried out with a three-dimensional image of a core sample from Mt. Simon sandstone, the target porous formation for a pilot CO_2_ sequestration project that is currently being carried out by Illinois State Geological Survey. Thus, no assumptions are made regarding the shape and sizes of the pore throats and pore bodies. The computed CO_2_ sorption isotherm at 195 K is in excellent agreement with our experimental data. The computed permeability is also in good agreement with the measurement. As a further test we also compute the sorption isotherm of N_2_ in the same formation at 77.3 K, and show that it is also in good agreement with our experimental data. The model is capable of predicting adsorption of CO_2_ (or any other gas for that matter) in porous formations at high pressures and temperatures. Thus, it is used to study the effect of hydrostatic pressure on adsorption and deformation of the porous formation under various conditions. We find that the effect of the confining pressure is more prominent at higher temperatures. Also computed is the depth-dependence of the capacity of the formation for CO_2_ adsorption, along with the induced volumetric strain.

## Introduction

Among the many methods that have been proposed for mitigating the effect of emission of CO_2_, its sequestration in geological formations is considered^1–9^ to be a promising way of addressing the problem of global warming. The geological formations of interest may include depleted oil and gas reservoirs, deep saline aquifers, and unminable coal seams^10–12^. In addition to its sequestration in depleted formations, the injected CO_2_ also provides sustained pressure that enhances the production of CH_4_, which competes with CO_2_^12,13^ for sorption on the internal surface of the reservoirs. Better understanding of the phenomenon entails having two main ingredients. One is the knowledge concerning the adsorption capacity of rocks. The second ingredient is a model of the formation itself.

Extensive studies of gas adsorption in geological formations have been carried out by both experiment^14–20^ and various theoretical and computational methods^21–28^. Grand-canonical Monte Carlo simulation^23,25^ has been the main computational approach for studying the sorption phenomenon, while such theoretical models as simplified local-density^16^ and the Ono-Kondo models^24,29,30^, as well as the density-functional theory^26,31^ constitute the main theoretical methods. In addition, molecular dynamics simulations^32^ have provided useful insight into the mechanism of gas sorption in microporous media. But, despite providing a basis for better understanding of gas sorption in porous media, almost all the theoretical and computational methods that have been utilized so far in order to study CO_2_ sorption in actual rock suffer from two main shortcomings. One is that models of the pore space that have been utilized are too simple, having practically no relation with the actual shapes and sizes of the pores in geological formations. For example, molecular simulation studies of CO_2_ sorption in rock have utilized a slit pore^25,33,34^, the space between two flat and parallel surfaces. The second shortcoming of the previous studies is the fact that experiments^13,35–37^ and limited computational studies^26,38^ have indicated that sorption of CO_2_ in rock may swell the formation, hence changing the structure of the pore space as adsorption proceeds. Adsorption-induced swelling of porous formations is a main factor that contributes to the change in the porosity, permeability, diffusivity, and surface area of a pore space^39^.

The main goal of the present paper is to address the aforementioned shortcomings. We describe a statistical mechanical model of gas sorption and the associated deformation in a porous medium. The model is utilized to compute the sorption isotherms and the deformation that it induces in a core sample from a sandstone reservoir - the Mount (Mt.) Simon formation. The formation is generally referred to the basal Cambrian formation in the upper Mississippi Valley and southern Great Lakes areas. Numerical simulation of the theoretical model is carried out using a three-dimensional (3D) image of a core sample from the formation. We then compare the results with our own experimental sorption isotherms and the permeability, measured with the same sample porous medium whose image we utilize in the simulations.

## Results and Discussion

### Estimating the parameters of the model

As Eqs (5) and (7) in the Methods section indicate, the Hamiltonian (total energy) of the system contains three parameters, namely, the fluid-fluid interaction energy parameter ${\epsilon }_{ff}$, the fluid-solid interaction energy parameter ${\epsilon }_{fs}$, and the coupling coefficient *λ* that links the deformation to sorption. One way of estimating ${\epsilon }_{ff}$ is through the mean-field theory that relates the bulk critical temperature *T*_*c*_ to ${\epsilon }_{ff}$ by, ${T}_{c}=Z{\epsilon }_{ff}/(4{k}_{B})$, where *k*_*B*_ is the Boltzmann’s constant. As is well-known, however, mean-field theories usually over- or underestimate various properties of fluid-solid systems. A more accurate way of estimating ${\epsilon }_{ff}$ and ${\epsilon }_{fs}$ is assuming that they are the energy interaction parameters that are used in molecular dynamics (MD) simulations. As Table 1 indicates, x-ray diffraction of the core samples of Mt. Simon shows that quartz - silica - constitutes about three-fourth of the sandstone’s composition. In fact, the quartz content of several core samples from the same location in Mt. Simon ranges from 73 percent to over 90 percent. Therefore, we use the Lennard-Jones (LJ) energy parameters for CO_2_ and CO_2_-silica^40,41^, ${\epsilon }_{{{\rm{CO}}}_{2}-{{\rm{CO}}}_{2}}/{k}_{B}=235.9$ K and ${\epsilon }_{{{\rm{CO}}}_{2}-{\rm{silica}}}/{k}_{B}=147.1$ K. In addition, since we also compute adsorption isotherm of nitrogen in the same core sample, we use the LJ parameters^40,41^, ${\epsilon }_{{{\rm{N}}}_{2}-{{\rm{N}}}_{2}}/{k}_{B}=94.45$ K, and ${\epsilon }_{{{\rm{N}}}_{2}-{\rm{silica}}}/{k}_{B}=147.3$ K.

**Table 1:** Mineralogy of the core samples according to their x-ray diffraction.

Whole-rock mineralogy	%
Quartz	73
K-feldspar	12
Plagioclase	7
Ankerite/Fe-dolomite	1
Fluorapatite	1
Pyrite	1
Clay	4
Clay	%
Illite-smectite	68
Illite	31
Chlorite	1
Kaolinite	1

In principle, the parameter *λ* can also be determined by MD simulations^21^. Here, however, we treat it as an adjustable parameter in order to fit the model to the data. Thus, *λ*, the model’s single fitting parameter, was estimated by fitting the calculated sorption isotherms to our experimental data for CO_2_ and N_2_ adsorption in the core samples at 195 K and 77.29 K, respectively. The optimal values of ${\lambda }^{\ast }=\lambda /{\epsilon }_{ff}$ were determined to be −40 and −30 for CO_2_ and N_2_, respectively. Negative values of λ^*^ imply that the porous medium swells as a result of gas adsorption.

As described in the Methods section below, we use the mean-field density-functional theory (DFT) to compute the contribution of the gas to the total energy of the system. As in any DFT formulation, one must estimate the maximum adsorption capacity, *n*_max_*v*_*p*_, with *n*_max_ and *v*_*p*_ being, respectively, the maximum density of the adsorbed phase, and the sorption pore volume. Their estimates are necessary because the gas density and the adsorbed amounts predicted by the DFT are in units of lattice site occupancy and, thus, must be converted to physical units. The minimum value of *n*_max_ is equal to reciprocal of the van der Waals co-volume, which for CO_2_ is 1.03 g/cm^3^ ^42^. The maximum value of *n*_max_ is the number density of a close packing of gas particles, assumed to be spherical, which is $\sqrt{2}/{\sigma }^{3}$ with *σ* being the molecular diameter of the gas, which for CO_2_ is 1.74 g/cm^3^. Since the maximum theoretical value of *n*_max_ agrees with the experimental data, we used it in our calculations. The corresponding value for N_2_ is 0.723 g/cm^3^. The sorption pore volume *v*_*p*_ is that of the core sample, computed based on the experimental values of the density and porosity of the sample whose sorption isotherm we have measured. The pore volume of the sample is 0.094 cm^3^/gr. The maximum adsorption capacity for CO_2_ and N_2_ are, respectively, 0.164 gr/gr and 0.068 gr/gr.

### The permeability of the core sample

Measurement of the gas permeability *k* of the core sample yielded, *k* = 325 millidarcy (mD). As described in the Modeling section, we computed the permeability of the core sample using its image together with the lattice-Boltzmann method. The result turned out to be, *k* = 368 mD, which differs from the experimental result by about 13 percent.

### Adsorption and volumetric strain isotherms

The absolute and excess adsorption were calculated by the usual equations,

1 $${\rho }_{{\rm{abs}}}=\frac{1}{M}\,\sum _{i}\,({\rho }_{i}),$$

2 $${\rho }_{{\rm{ex}}}=\frac{1}{M}\,\sum _{i}\,({\rho }_{i}-{\rho }_{b}),$$

where *M* is the total number of grid sites in the pore space, and *ρ*_*b*_ is the density of the bulk fluid. The computations were carried out as a function of the pressure *P*. The bulk density *ρ*_*b*_ used in the DFT is in units of lattice site occupancy and is related to the physical bulk density *ρ*_*p*_ through, *ρ*_*b*_ = *ρ*_*p*_/*n*_max_. To estimate *ρ*_*p*_, one may use either the experimental data or an equation of state. Since the Peng-Robinson equation of state^43^ provides^44^ accurate predictions for the density of gases over a wide range of pressure, we utilized it for calculating *ρ*_*p*_. The bulk chemical potential *μ*_*b*_ is given by the standard mean-field equation^45^

3 $${\mu }_{b}={k}_{B}T\,\mathrm{ln}\,(\frac{{\rho }_{b}}{1-{\rho }_{b}})-Z{\epsilon }_{ff}{\rho }_{b},$$

where *Z* is the coordination number. In the model *Z* = 4, as we used tetrahedral grid blocks.

A series of bulk pressures *P* were selected and the corresponding bulk density *ρ*_*p*_ were calculated using the Peng-Robinson equation of state. Then, the corresponding bulk DFT densities, *ρ*_*b*_ = *ρ*_*p*_/*n*_max_ were computed, and Eq. (3) was used to compute the bulk chemical potential *μ*_*b*_. The results were then used in Eqs (8) and (15) (see the Methods section below) in order to compute the sorption isotherms along with the mechanical response of the porous solid. The bulk pressure does, of course, exert an equivalent external force on the boundaries of the porous sample, which is utilized in the calculation of the volumetric strain via the finite-element formulation that is described below in the Methods section.

**Figure 1:**
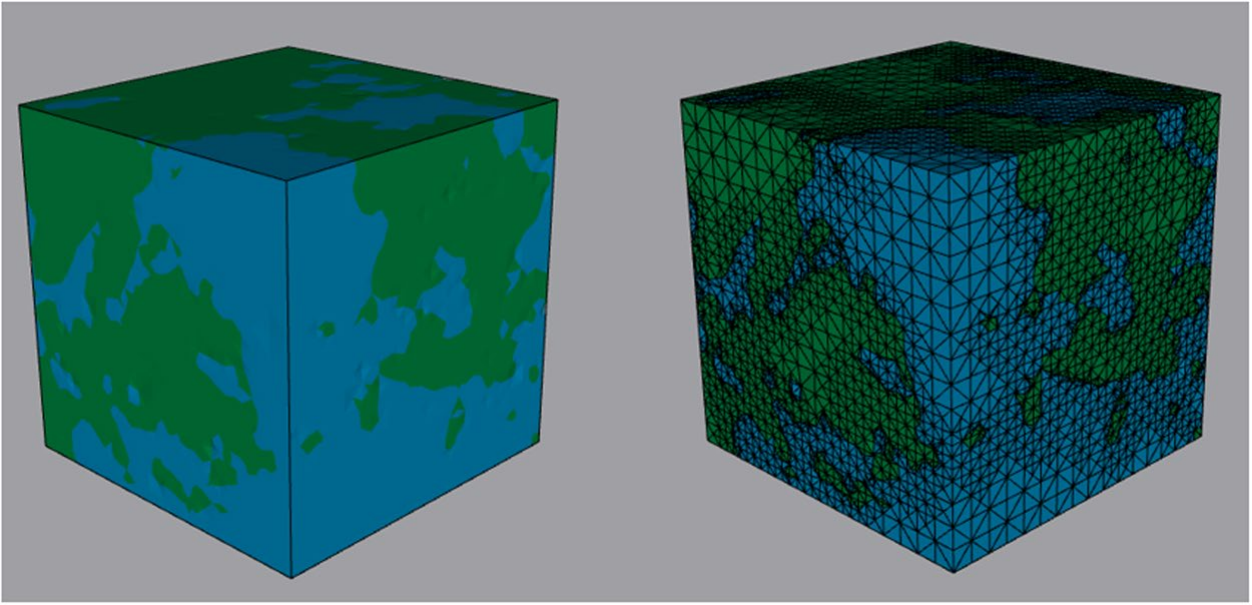
Image of the sandstone sample (left) and its discretization with adaptive tetrahedral mesh. Green represents the pore space, while blue indicates the solid skeleton. The image was segmented and discretized using Seg3D 2.4.0^63^ and Cleaver 2.2^64^, respectively.

Figure 1 presents the 3D image of the porous medium and its grid structure used in the computations. The shear modulus *G* and the Poisson’s ratio *ν* of the solid matrix of the core sample were taken to be 2.73 GPa and 0.26, respectively, which are the typical experimental values reported for sandstone.

**Figure 2:**
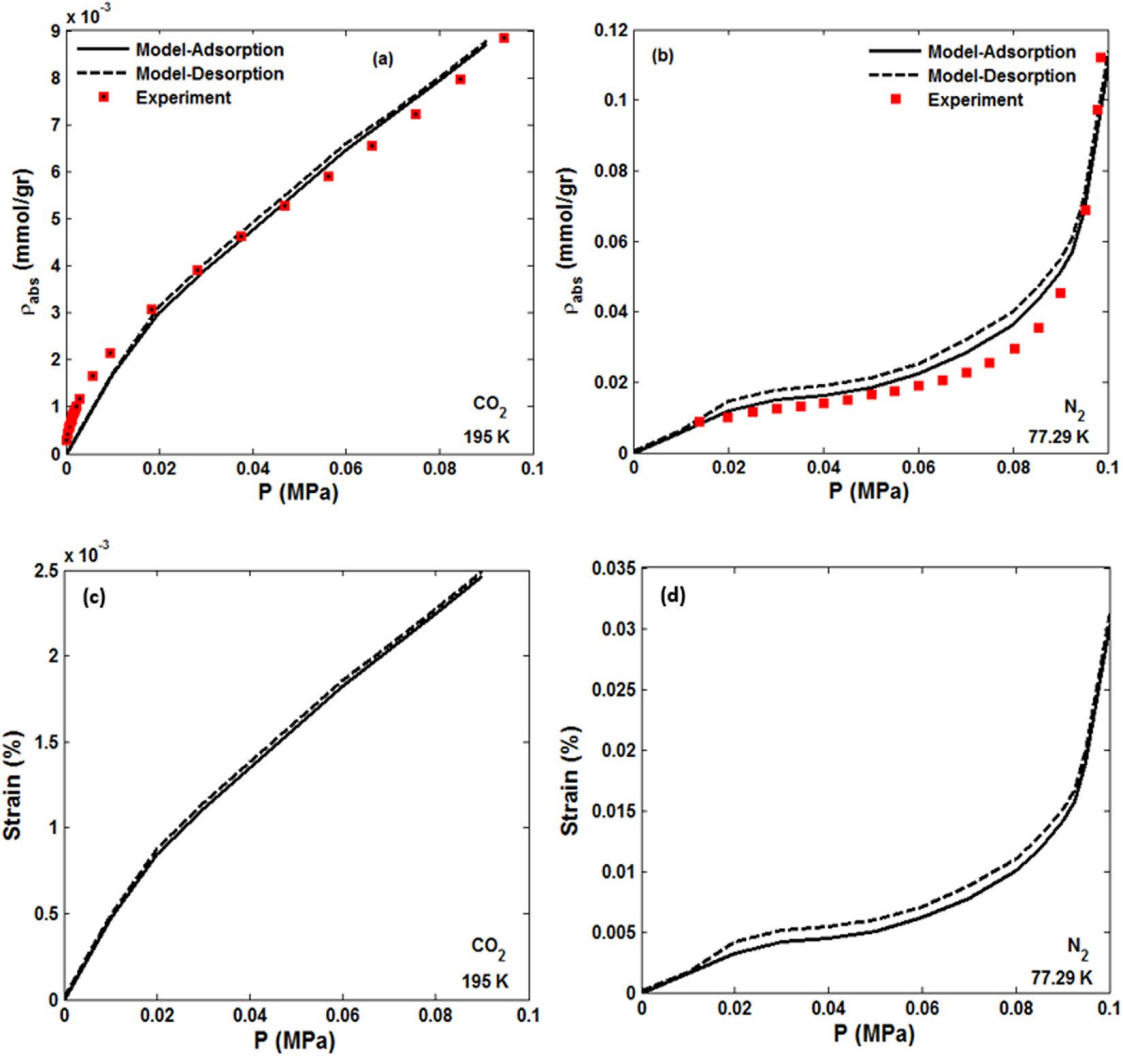
Comparison of the experimental data with the computed adsorption isotherms. Also shown are the computed desorption isotherms. The corresponding computed dependence of the volumetric strains on the pressure are shown in (**c**,**d**).

The computed sorption isotherms are shown in Fig. 2, where they are compared with our experimental data. It is clear that the data and the computed isotherms agree closely. Also shown are the computed desorption isotherms in the same core sample. The difference between adsorption and desorption isotherms for both gases is small, although it is a bit larger in the case of N_2_. According to the classification of the International Union of Pure and Applied Chemistry^46^ (IUPAC), the sorption isotherm of N_2_ is of Type III, which corresponds to a case in which attractive adsorbent-adsorbate interactions are weak, but molecular interactions between the adsorbates themselves are strong. On the other hand, although Fig. 2 does not indicate it because the calculations and measurements shown in the figure are restricted to low pressures, in the IUPAC classification the CO_2_ sorption isotherm corresponds to Type I, which occurs when adsorption is limited to at most a few molecular layers, so that with increasing pressure the amount adsorbed reaches a constant value and does not change at still higher pressures. This is demonstrated shortly.

The volumetric response of the solid matrix of the porous medium represents its adsorption-induced swelling and elastic compression arising due to the fluid pressure. Interestingly, the volumetric strain “isotherms” in the solid matrix of the core sample, calculated as the sum of the principal strains, corresponds closely to the sorption isotherms of the two gases. This is also shown in Fig. 2, where we present the pressure-dependence of the strain during sorption of the two gases. The deformation of the rock due to adsorption of the gases is small, because the temperature is very low and the core sample is mostly quartz, which is very difficult to deform.

**Figure 3:**
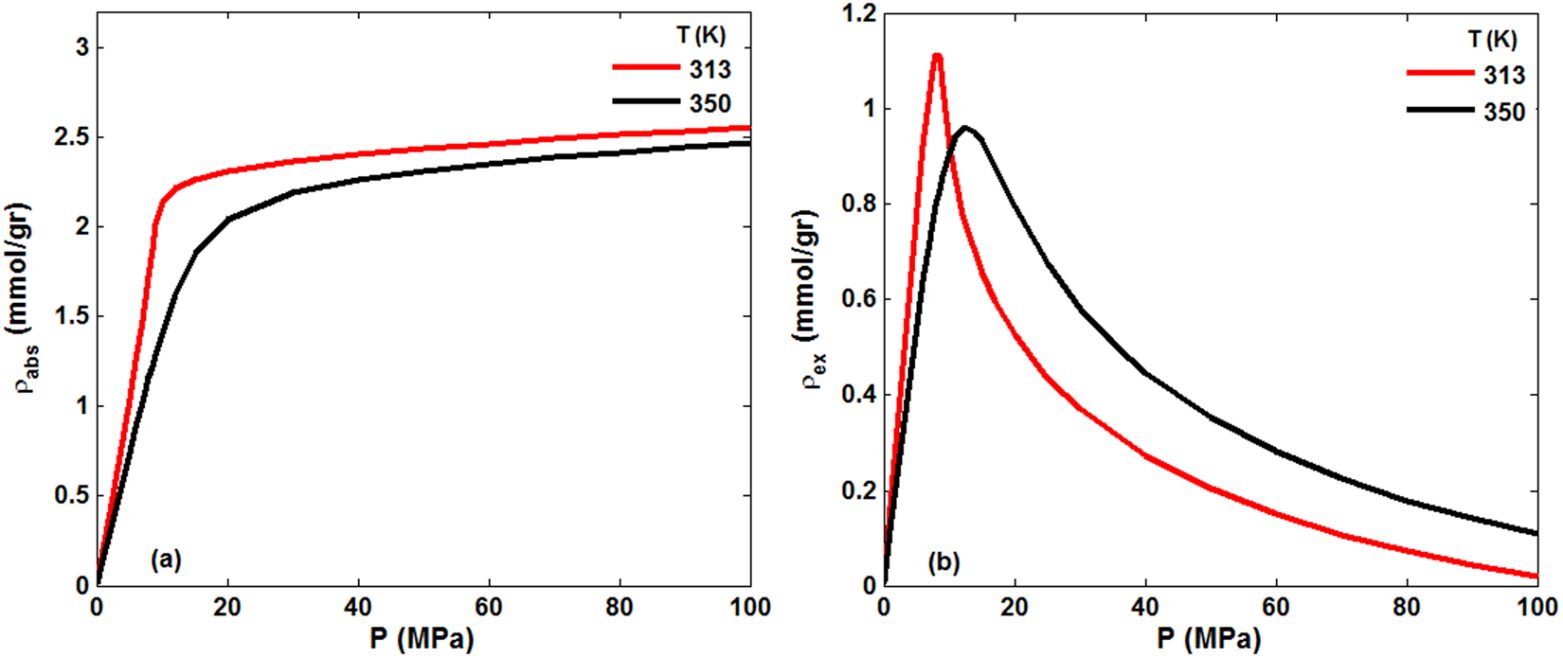
CO_2_ (**a**) absolute and (**b**) excess adsorption in the porous sample at 313 K and 350 K. No hydrostatic pressure was applied to the structure.

Since the focus of the work is sequestration of CO_2_ in rock, hereafter we present the results only for CO_2_. Thus, we simulated sorption of CO_2_ in the core sample at relatively high temperatures of 313 K and 350 K and a wide range of pressures that are relevant to the geological conditions for CO_2_ sequestration in Mt. Simon. Figure 3 presents the absolute and excess adsorption uptakes of CO_2_ at the two temperatures, demonstrating that the isotherms are indeed of the aforementioned Type I. The excess adsorption attains a maximum, followed by a decreasing trend at higher pressures. The reason for the trend is that, at pressures below the maximum the gas densities in the pore space and in the bulk both increase with the same rate. At pressures higher than the pressure at which the maxima are attained, however, the pore space is saturated and, thus, the adsorbed amount no longer change^47,48^. The smaller magnitude of the maximum at the higher temperature and its shift to a higher pressure are, of course, expected. At low pressures the differences between the two isotherms are negligible, consistent with the previous experimental and theoretical studies of CO_2_ adsorption in shale and coal formations^22,49^.

**Figure 4:**
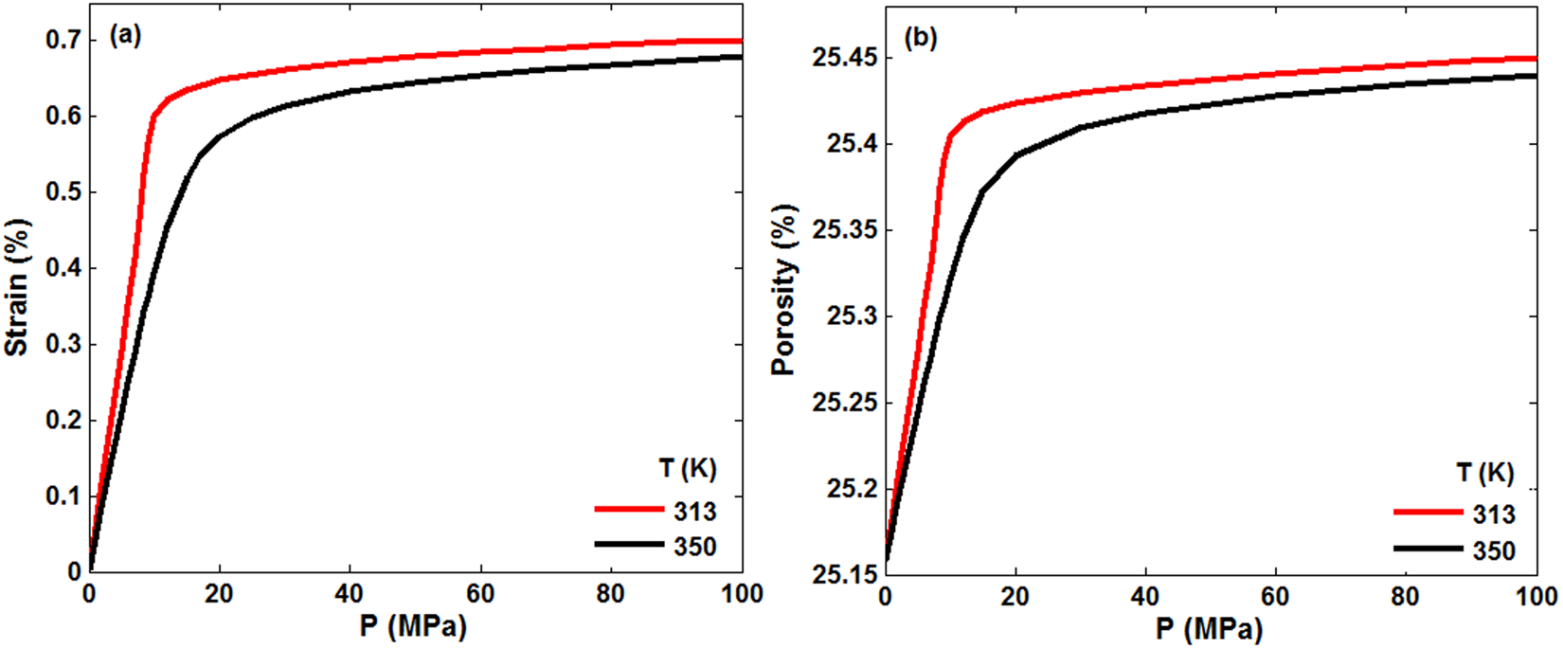
(**a**) Volumetric strain and (**b**) the porosity, corresponding to the isotherms of Fig. 3, at 313 K and 350 K.

The volumetric strain ${\epsilon }_{V}$ and porosity change, induced by CO_2_ adsorption in the porous formation at 313 K and 350 K, which correspond to Fig. 3 are depicted in Fig. 4. ${\epsilon }_{V}$ is dependent upon the temperature and is higher at lower temperatures, because the amount of gas adsorbed is higher at low *T*. The strain “isotherm” follows a pattern similar to sorption, namely, it increases rapidly for pressures up to 10 MPa at 313 K and 15 MPa at 350 K, whereas at still higher pressures the strain changes only slightly, because the pore space has been saturated by CO_2_. Similar trends were reported for CO_2_ adsorption in coalbeds^50,51^. The maximum ${\epsilon }_{V}$ due to the adsorption is 0.7% and 0.68% for 313 K and 350 K, respectively, in line with the experimental data for CO_2_ adsorption on coalbeds^49^. Note that the pressure at which the strain reaches its maximum corresponds to that at which maximum adsorption is obtained. On the other hand, the porosity increase of the porous formation induced by CO_2_ adsorption is only about 1.15% and 1.11% at 313 K and 350 K, respectively. The small change in the porosity is, once again, reflective of the fact that the core sample is largely quartz and very difficult to deform.

### Effect of the hydrostatic stress

**Figure 5:**
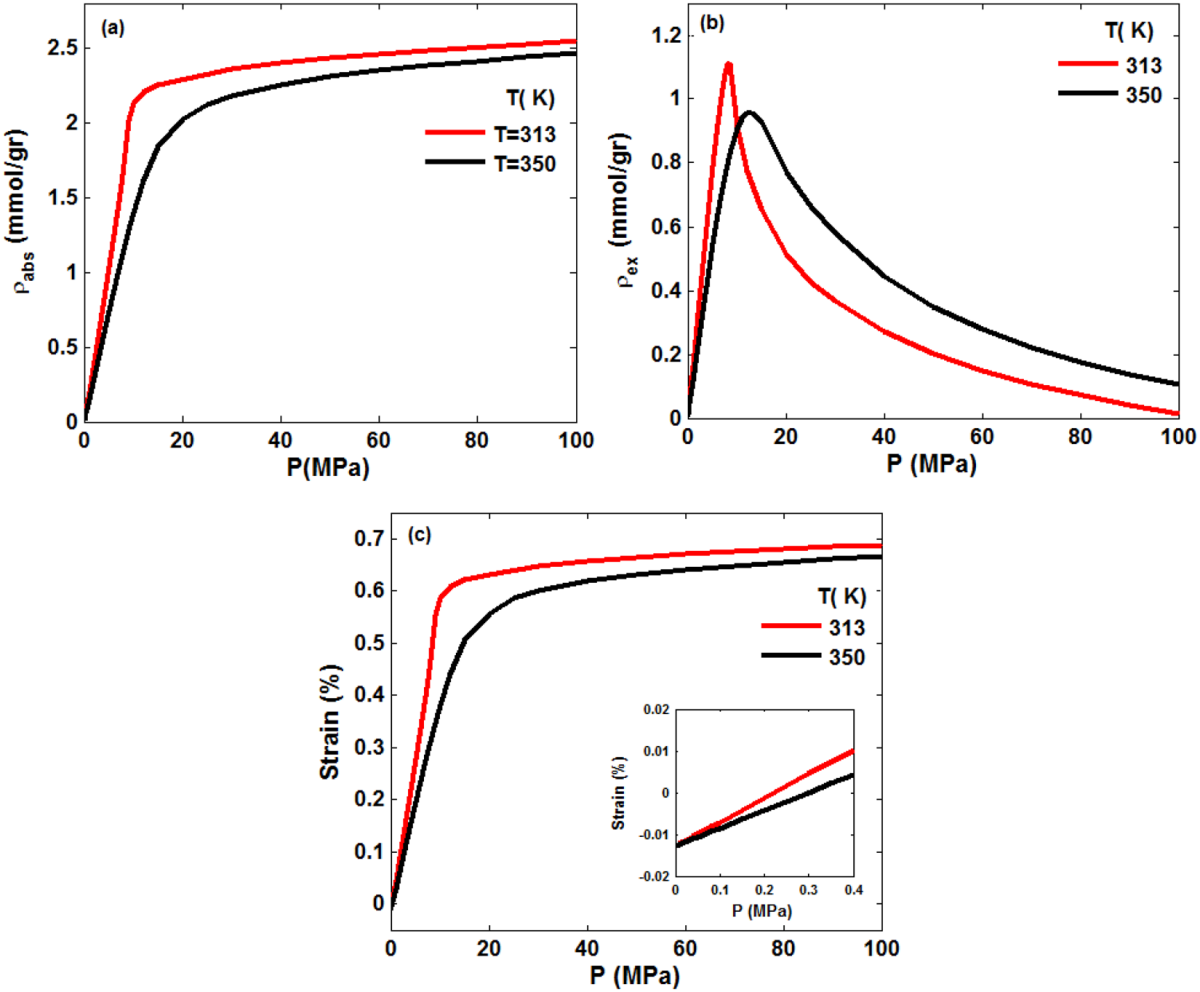
Absolute and excess adsorption, as well as the volumetric strain in the porous medium under a hydrostatic pressure of 50 MPa and two temperatures.

Since the optimal sites for CO_2_ sequestration are located deep within an underground porous formation, it is essential to consider the effect of external confining stress on the adsorption and deformation. Thus, we computed adsorption isotherms and volumetric strains for confining pressure of 50 and 100 MPa. In other word, in addition to the pressure of the gas in the bulk phase, the porous medium experiences an excess stress from the hydrostatic pressure. The results for the hydrostatic pressure of 50 MPa are shown in Fig. 5. The maximum values of the excess adsorption at 313 K and 350 K are, respectively, 1.11 mmol/gr and 0.95 mmol/gr, while the corresponding volumetric strains are 0.69% and 0.67%. These should be compared with the results shown in Fig. 4. The inset of Fig. 5(c) indicates negative strain, or solid contraction, at low pressures, followed by increasing positive strain, indicating swelling. Thus, at low pressures the stress arising from the confining pressure dominates the effect of swelling induced by adsorption. The effect is more prominent at higher temperatures. But, at high pressures, it is adsorption-induced swelling that is more effective than the hydrostatic compression.

**Figure 6:**
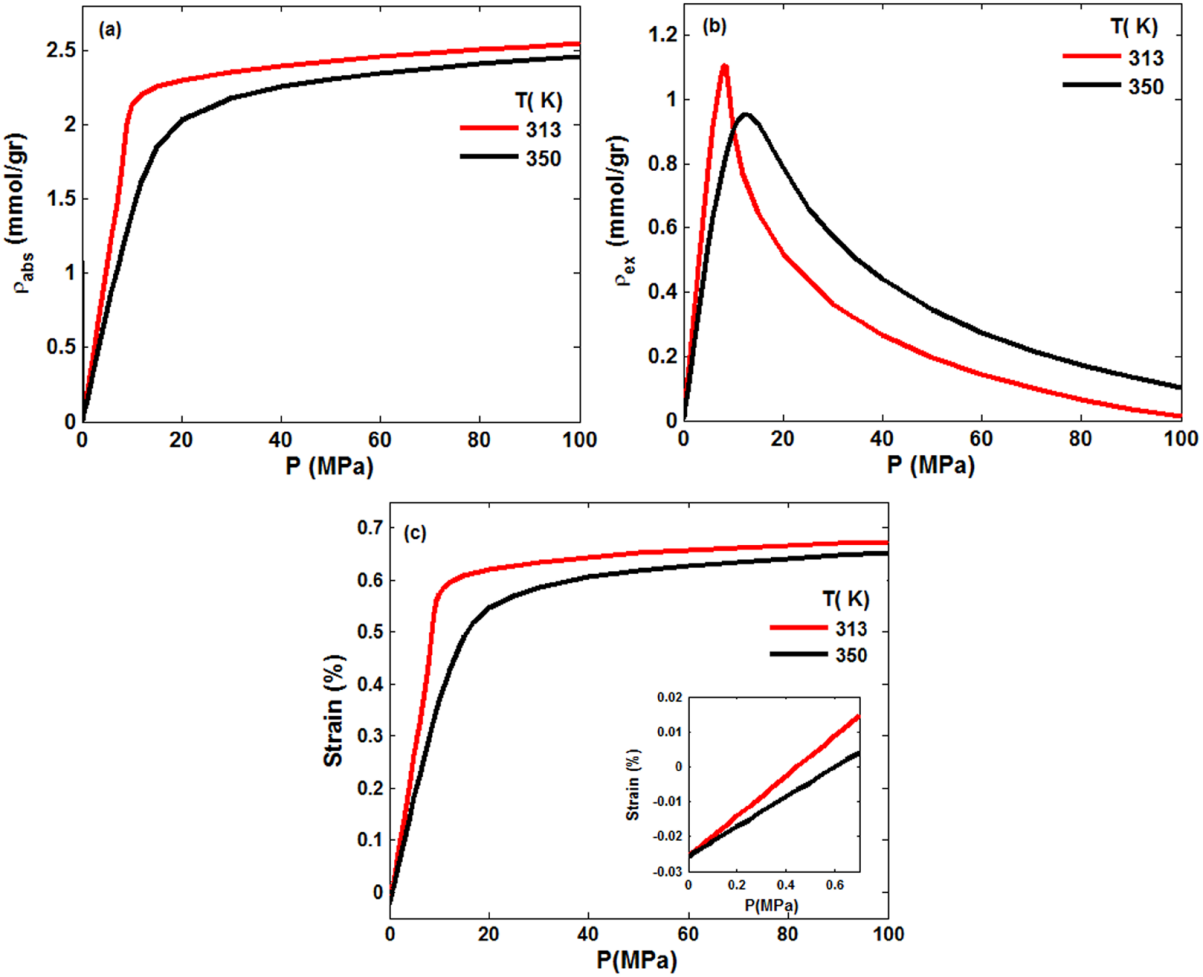
Same as in Fig. 5, except that the hydrostatic pressure is 100 MPa.

**Figure 7:**
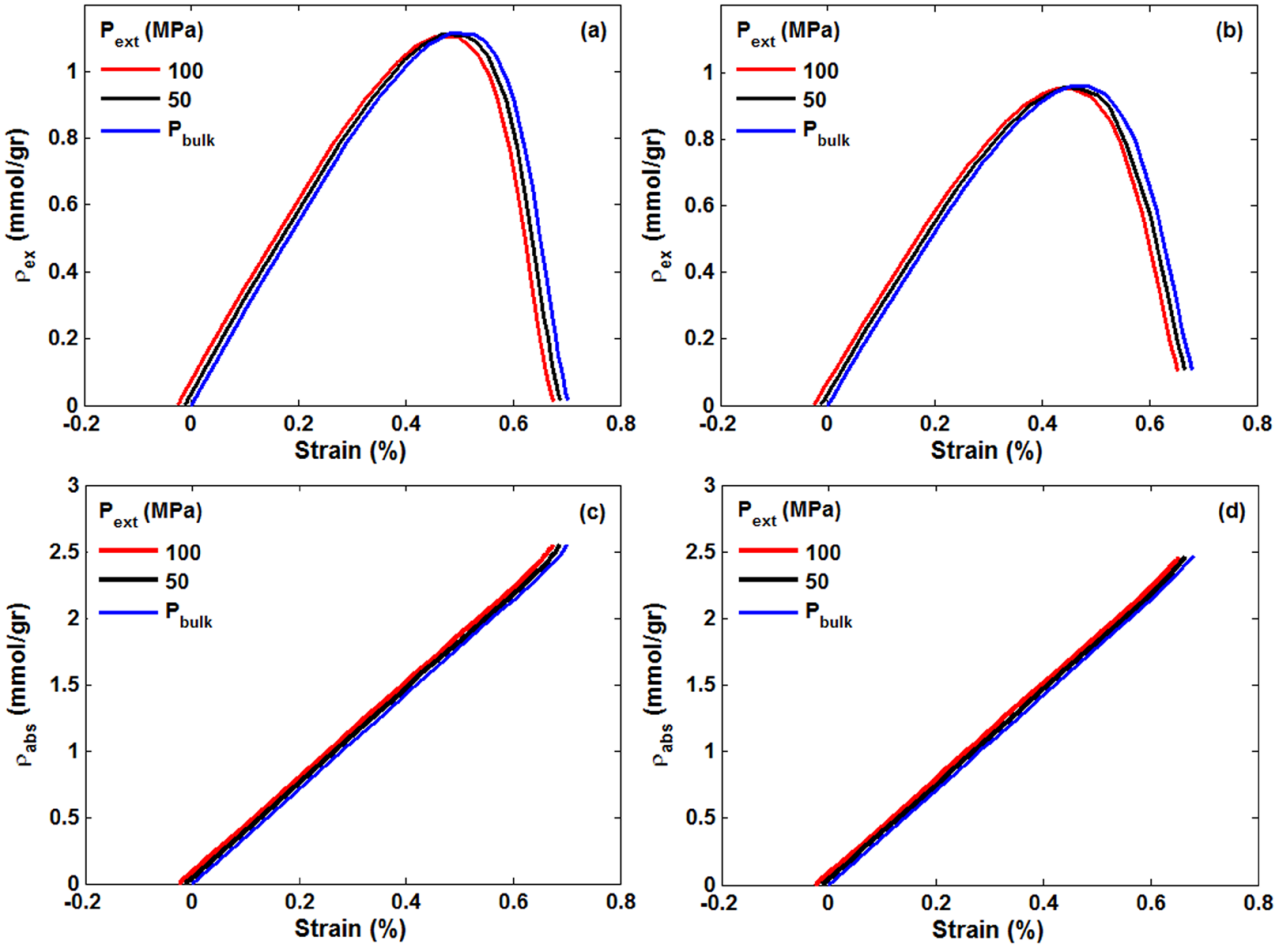
Strain-dependence of CO_2_ excess and absolute adsorption in the porous medium under three hydrostatic pressures and temperatures of (**a**,**c**) 313 K and (**b**,**d**) 350 K.

The results for a hydrostatic pressure of 100 MPa are shown in Fig. 6. They demonstrate patterns similar to those shown in Fig. 5, with only slight changes in the numerical values of the maximum excess adsorption and the volumetric strain, and with the difference that the contraction-dominated stage occurs over a broader pressure range and higher compressive strains. To better understand the effect of the external pressure, we compare in Fig. 7 the strain-dependence of the absolute and excess amounts of sorbed CO_2_ versus volumetric strain at 313 K and 350 K and for three hydrostatic pressures.

### Adsorption under geological conditions

**Figure 8:**
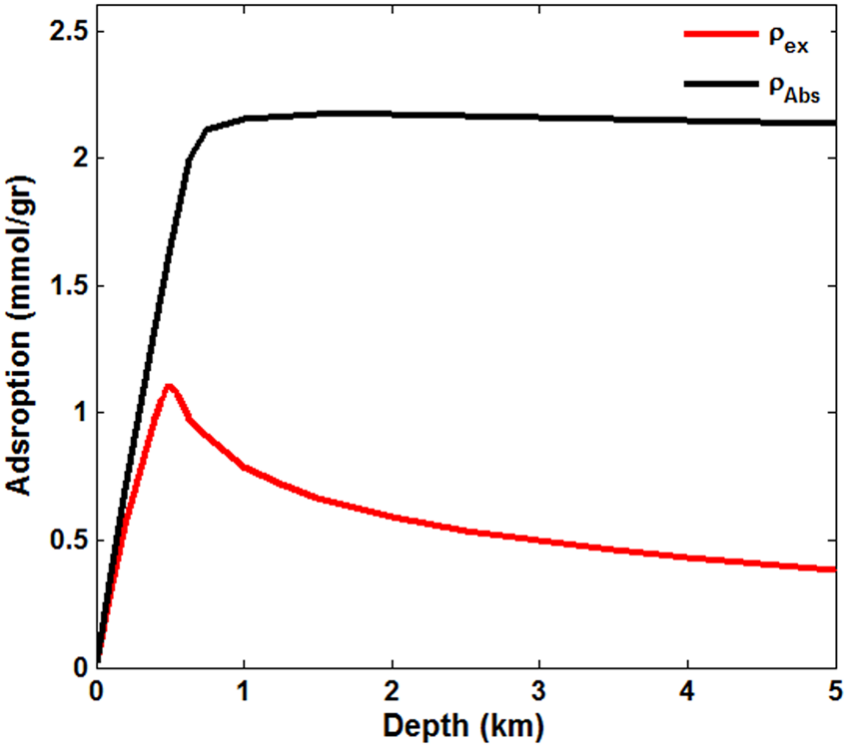
Dependence on the depth of the (maximum) absolute and excess adsorption of CO_2_ with a hydrostatic pressure gradient of 15 MPa/km and temperature gradient of 27.3 °C/km.

Due to the variations of temperature and pressure with depth in geological formations, their effect on the adsorbed amounts of gases cannot be neglected. To this end, we assumed that the pressure and temperature are linear functions of the depth, using a pressure gradient of 15 MPa/km and temperature gradient of 27.3 °C/km^52^. We then computed the depth-dependence of the maximum adsorption capacity and volumetric strain for CO_2_. The results for sorption are presented in Fig. 8. In shallower depths, up to about 200 m, the difference between the excess and absolute adsorption is negligible. The shapes of the two curves are also similar to what we have presented so far, namely, that the excess amount of adsorbed CO_2_ rapidly increases to a maximum value at small depths, followed by a gradual decrease at larger depths. The depth-dependence of the absolute adsorption is also similar to what we have presented so far: It increases sharply at small depths, and then remains essentially unchanged in the samples that are at larger depths. Both sets of results are consistent with the available experimental data for CO_2_ sorption in shale and coal^26,52^. The results shown in Fig. 8 and their comparison with the results presented so far suggest that, although high temperatures in deep underground formations adversely affect the maximum adsorption capacity, the effect of high pressures at the same depth is strong enough to offset the unfavorable thermal effect.

## Summary

We presented a model for sorption-induced deformation of porous formations that is capable of predicting the sorption isotherms in the formations. The model was used to study CO_2_ adsorption in sandstone, along with the associated deformation that it induces in the porous sample. The computations utilized the 3D image of the core sample from Mt. Simon sandstone and, therefore, no assumption was made regarding the shapes and sizes of the pore bodies and pore throats of the pore space. The model’s predictions for low-temperature sorption isotherms of CO_2_ and N_2_ are in good agreement with our experimental data. A study of CO_2_ adsorption in the porous medium under a hydrostatic pressure indicated stress-induced compression in the solid matrix at low pressures, followed by swelling at high pressures. T

The model is capable of predicting sorption isotherms and the resulting deformation in deep subsurface porous formations, and indicates that in such formations the excess adsorption quickly reaches a maximum, beyond which it gradually decreases, with absolute adsorption and volumetric strain following the same trends with the depth.

## Methods

### Measurement of the sorption isotherm

Gas adsorption in two cylindrical core samples from Mt. Simon formation were measured. The samples are both horizontal plugs from a larger section that had been extracted along a well in the formation. More specifically, sample 1 was from a depth of 6979.57 ft, whereas sample 2 was from a depth of 6985.85 ft. Their dimensions and weights are listed in Table 2. The mineralogy of the parent core sample from which the two cores were extracted is presented in Table 1. Prior to the sorption measurements, the samples’ porosities were measured via the helium expansion method. They were virtually identical, and are listed in Table 2.

**Table 2:** Properties of the core samples.

Core	Depth (ft)	L (cm)	D (cm)	W (g)	Porosity (%)
1	6979.6	4.88	2.51	46.05	24.8
2	6985.6	5.16	2.47	49.04	24.9

Before sorption experiments began, the samples were dried at 110 °C under vacuum (1 mm Hg) for 24 hours. The N_2_ and CO_2_ adsorption isotherms were measured using a Micromeritics ASAP 2010 Instrument. The N_2_ sorption test was carried out at the nominal temperature of 77.3 K, which was maintained by immersing the specialty-made sample-holder containing the core sample in a liquid-nitrogen bath. Note, however, that the true adsorption temperature varies a bit during the experiment. The instrument, however, measures the true experimental temperature and calculates the corresponding N_2_ saturation pressure. The CO_2_ adsorption measurements were carried out at 195 K, which was maintained by immersing the sample-holder containing the core in a bath containing a mixture of dry ice and acetone. To generate the adsorption isotherm, the pressure in the sorption cell was increased in a step-wise manner, and the adsorbed gas volume was measured and recorded. The data will be compared with the predictions of the model (see the Results and Discussion section).

### The model

Assuming that the sorption-induced swelling is slow and quasi-static, we use equilibrium statistical mechanics to formulate a model that describes sorption and its effect on the deformation and swelling of the pore space. The total energy *E* of the system - the porous formation and the gas - is given by

4 $$E={E}_{f}+{E}_{s}+{E}_{fs},$$

where *E*_*f*_, *E*_*s*_, and *E*_*fs*_ represent, respectively, the energy due to the fluid, the solid, and the interactions between the two. The energy of the fluid is expressed by a mean-field DFT^21,53–55^,

5 $${E}_{f}={k}_{B}T\,\sum _{i}\,[{\rho }_{i}\,\mathrm{ln}\,{\rho }_{i}+(1-{\rho }_{i})\,\mathrm{ln}(1-{\rho }_{i})]-\frac{1}{2}{\epsilon }_{ff}\,\sum _{i}\,\sum _{Z}\,{\rho }_{i}{\rho }_{i+Z}-\mu \,\sum _{i}\,{\rho }_{i},$$

where *ρ*_*i*_ is the average fluid density (average occupancy) at lattice site *i*, *μ* is the chemical potential of the bulk fluid, and ${\epsilon }_{ff}$ is the interaction parameter between neighboring fluid sites. Equation (5) arises from a lattice-gas model in which only the nearest-neighbor interactions are taken into account. The density of each lattice site *i* is determined by considering two indicator functions *I*_*i*_ and ${ {\mathcal I} }_{i}$ for each site, such that ${I}_{i}^{(f)}=0$ and 1 and ${I}_{i}^{(s)}=1$ and 0 denote, respectively, the fluid and the solid indicators. Then, the density at each site is given by, ${\rho }_{i}=\langle {I}_{i}^{(f)}[1-{I}_{i}^{(s)}]\rangle$, where 〈·〉 indicates the average value. We assume that the solid matrix of the porous formation is linearly elastic (an assumption that can be relaxed, provided that one has a constitutive equation that expresses the elastic properties of the solid^56,57^). Then, *E*_*s*_, the energy of the solid is represented by

6 $${E}_{s}=\frac{1}{2}\,\sum _{i}\,[{K}_{i}{({\boldsymbol{\nabla }}\cdot {{\bf{u}}}_{i})}^{2}+2{\sigma }_{i}({\boldsymbol{\nabla }}\cdot {{\bf{u}}}_{i})],$$

where *K*_*i*_ is the elastic stiffness, and *σ*_*i*_ and ${({\boldsymbol{\nabla }}\cdot {\bf{u}})}_{i}$ are the stress and strain in element *i*, respectively. The energy *E*_*fs*_ associated with the fluid-solid interactions is the sum of wetting energy *E*_*w*_ at the fluid-solid interface, plus the energy change *E*_*d*_ in the solid as a result of its sorption-induced deformation/swelling^21,58^:

7 $${E}_{fs}\equiv {E}_{w}+{E}_{d}=-\,\frac{1}{2}{\epsilon }_{fs}\,\sum _{i}\,{\rho }_{i}+\frac{1}{2}\lambda \,\sum _{i}\,({\rho }_{i}\,{\boldsymbol{\nabla }}\cdot {{\bf{u}}}_{i}).$$

Here, **u**_*i*_ is the displacement vector at *i*, ${\epsilon }_{fs}$ is the wettability coefficient that measures the strength of the interaction between fluid and solid, and *λ* is the coupling constant that relates the variation in the strain energy of the solid (i.e., its deformation) due to the fluid and solid phases. The sum runs over all the lattice sites. To obtain the equilibrium state of the porous medium and its fluid content, which is in contact with a bulk reservoir at temperature *T* and chemical potential *μ*, we minimize the total energy with respect to the fluid density and strain **u**_*i*_ of the solid. This completes the formulation of the model.

### Numerical simulation of sorption and deformation

We used the finite-element method to solve the governing equations. Minimizing the total energy with respect to *ρ*_*i*_ and **u**_*i*_ yields two equations, one for the local fluid density *ρ*_*i*_, and a second one for the displacement field of the solid. The two equations are coupled and must be solved numerically. The governing equation for density of fluid *ρ*_*i*_ is given by,

8 $${\rho }_{i}={\{1+\exp [-\beta (\mu +{\epsilon }_{fs}+{\epsilon }_{ff}\sum _{Z}{\rho }_{i+Z}-\lambda {\boldsymbol{\nabla }}\cdot {{\bf{u}}}_{i})]\}}^{-1},$$

whereas the displacement **u**_*l*_ of vertex *l* in an element *i* of the computational grid is governed by

9 $${{\bf{k}}}_{e}{{\bf{u}}}_{l}=\frac{1}{2}{{\bf{e}}}_{ni}\lambda {\rho }_{i}.$$

In Eq. (9) **e**_*ni*_ are the unit vectors from the center of the element *i* to its vertices *n*, and **k**_*e*_ is the elemental stiffness matrix given by

10 $${{\bf{k}}}_{e}={\int }_{V}\,{{\bf{U}}}^{{\rm{T}}}{\bf{C}}{\bf{U}}\,dV,$$

where **C** and **U** are, respectively, the elastic coefficient and the strain-displacement matrices, and T denotes the transpose operation. The integration is over the volume *V* of the element in the computational grid. **C** is given by,

11 $$C=(\begin{array}{cccccc}\lambda ^{\prime} +2\mu ^{\prime}  & \lambda ^{\prime}  & \lambda ^{\prime}  & 0 & 0 & 0\\ \lambda ^{\prime}  & \lambda ^{\prime} +2\mu ^{\prime}  & \lambda ^{\prime}  & 0 & 0 & 0\\ \lambda ^{\prime}  & \lambda ^{\prime}  & \lambda ^{\prime} +2\mu ^{\prime}  & 0 & 0 & 0\\ 0 & 0 & 0 & \mu ^{\prime}  & 0 & 0\\ 0 & 0 & 0 & 0 & \mu ^{\prime}  & 0\\ 0 & 0 & 0 & 0 & 0 & \mu ^{\prime} \end{array}),$$

where *λ*′ and *μ*′ are the Lamé constants that are related to the shear modulus *G* and the Poisson’s ratio *ν*,

12 $$\lambda ^{\prime} =\frac{2G\nu }{1-2\nu },$$

13 $$\mu ^{\prime} =G.$$

The 3D computational grid that we utilize consists of tetrahedron grid cells; see Fig. 1. We first introduce three normalized coordinates (*ξ*, *η*, *ζ*) ≡ (*f*_1_, *f*_2_, *f*_3_) that are related to the Cartesian coordinates (*x*, *y*, *z*) by, $x={x}_{1}+{\sum }_{i=2}^{4}\,({x}_{i}-{x}_{1}){f}_{i-1}$, $y={y}_{1}+{\sum }_{i=2}^{4}\,({y}_{i}-{y}_{1}){f}_{i-1}$, and $z={z}_{1}+{\sum }_{i=2}^{4}\,({z}_{i}-{z}_{1}){f}_{i-1}$, with (*x*_*i*_, *y*_*i*_, *z*_*i*_) (*i* = 1 − 4) being the coordinates of the vertices of the tetrahedra. We then introduce a shape vector, **N** = (*N*_1_, *N*_2_, *N*_3_, *N*_4_)^T^ whose components *N*_*i*_(*ξ*, *η*, *ζ*) are, ${N}_{1}=1-\sum \,j={2}^{4}{f}_{j-1}$, and *N*_*j*_ = *f*_*j*−1_ with *j* = 2 − 4. In Eq. (10) **U** is a 3 × 4 matrix given by

14 $${\bf{U}}={\bf{D}}{\bf{N}},$$

where **D** is the differentiation operator matrix, so that the entries *U*_*ij*_ of **U** are given by, *U*_*ij*_ = ∂*N*_*i*_/∂*f*_*j*_. A global stiffness matrix **K** is then constructed by compiling in it the stiffness matrices of the tetrahedral elements. Doing so generates the following system of linear equations that describe the displacements of elemental vertices:

15 $$\sum _{l}\,{K}_{nl}{{\bf{u}}}_{l}=\frac{1}{2}\,\sum _{i}\,{{\bf{e}}}_{ni}\lambda {\rho }_{i}.$$

Equations (8) and (15) must then be simultaneously solved numerically.

We compute the sorption isotherms of CO_2_ and N_2_ and the deformation that they induce in the 3D core sample from Mt. Simon sandstone, whose actual 3D image of the sample^59^, shown in Fig. 1(a), we utilize in the simulations. Due to the limitations on the resolution of the image, the porosity of the image is 10 percent. Thus, based on the correlation function of the pore sizes^60,61^ we generated pores with sizes below the resolution of the image and distributed them in the image, so that its total porosity matched that of the core sample.

To carry out the computations, we must first discretize the 3D image in order to generate the computational grid. To do so, one must first determine the size of the representative elementary volume (REV) for the sample, i.e., the minimum image size such that its properties will not change if larger images and computational grids are used. In a previous study^59^ in which flow of CO_2_ and water in the same image was simulated, it was determined that an image with 300 × 300 × 300 voxels represents adequately the REV. Thus, we used the same image size in the present study.

The computational grid must be resolved enough to accurately represent the image’s morphology, but it must also be such that the required computations do not take prohibitively long times. We discretized the domain with an adaptive grid with tetrahedral elements, shown in Fig. 1(b). After some preliminary simulations in which various grid resolutions were utilized in order to identify the most accurate grid with affordable computational time, a grid with 373,607 tetrahedral elements was determined to be accurate enough, as the porosity and computed adsorption isotherm of CO_2_ did not change significantly if grids with higher resolutions were used.

The numerical procedure for determining the fluid density and displacement fields is as follows. We begin with a given chemical potential *μ* and initial guesses for the density *ρ*_*i*_ (such as a uniform profile across the sample) and ${\boldsymbol{\nabla }}\cdot {{\bf{u}}}_{i}$, and substitute them in the right side of Eq. (8), which provides a new approximation for *ρ*_*i*_. The new approximate *ρ*_*i*_ is then substituted in the set of equation (15) and the set is solved by the generalized minimal residual method to obtain a new approximate solution for the displacement field. The new approximations are then substituted in the right side of Eq. (8) to obtain the next approximate solution for *ρ*_*i*_. We then return to Eq. (15) to calculate the new displacement field. The iteration continues until constant density and displacement fields are obtained.

### Numerical calculation of the effective permeability

We utilized the lattice-Boltzmann (LB) method with a single relaxation time to simulate fluid flow in the pore space of the image. The effective permeability was computed using Darcy’s law. Using the standard bounce-back rule, the no-slip boundary condition was imposed on the internal solid boundaries. A constant pressure gradient was applied in the flow direction. The D3Q19 propagation scheme^62^ was used in LB method in order to evolve the flow system.

The LB model is second-order accurate, with its compressibility error related to the square of the Mach number, Ma. In order to simulate an incompressible fluid, Ma is kept below 0.1. A unit relaxation parameter *τ*_*R*_ = 1 of the LB method was used, because it leads to accurate simulation of fluid flow^62^. The simulations were carried out in the creeping flow regime with a Reynolds numbers, Re < 1. Some preliminary simulations were carried out to determine an accurate grid resolution and size. We determined that a grid of size 300 × 300 × 300 yields accurate estimate of the permeability with reasonable computation time.
